# Platelet alloimmunization in transfusion-dependent thalassemia patients from Southern China (2014-2023)

**DOI:** 10.3389/fimmu.2025.1687913

**Published:** 2025-10-03

**Authors:** Yuchen Huang, Yuxi Chen, Qiuhong Mo, Huihui Mo, Fang Lu, Hengcong Li, Lilan Li, Jierun Chen, Lihong Jiang, Yan Zhou, Zhoulin Zhong

**Affiliations:** Nanning Blood Center, Nanning, China

**Keywords:** transfusion-dependent thalassemia (TDT), platelet antibody, specificity of antibody, transfusion, cross-matching

## Abstract

Patients with transfusion-dependent thalassemia (TDT) are at high risk of alloimmunization. While previous research is predominantly focused on red blood cell alloimmunization, the potential risks of platelet alloimmunization are frequently underestimated. We conducted a retrospective study of 249 TDT patients who underwent platelet immunology testing between January 2014 and December 2023 at Nanning Blood Center. Flow cytometry was used for platelet antibody screening, followed by enzyme-linked immunosorbent assay for detailed platelet antibody identification. Among the 249 TDT patients, 103 were detected positive for platelet alloimmunization (41.37%). Patients older than 12 had a higher prevalence of platelet alloimmunization than those younger than 12 (54.17% vs. 36.16%, *P=*0.009). Most of the platelet antibodies were human leukocyte antigen (HLA) antibodies alone (79.61%). Two TDT patients received cross-matched platelets and demonstrated successful transfusion responses with 24-hour corrected count increment being 10.62 and 7.80, respectively.TDT patients demonstrate a high prevalence of platelet alloimmunization, characterized by a diverse array of antibodies, predominantly HLA antibodies. In conclusion, proactive screening for platelet antibodies and employing cross-matched platelet transfusions can mitigate alloimmunization risks in TDT patients and address the clinical challenge of platelet transfusion refractoriness.

## Introduction

Transfusion-dependent thalassemia (TDT) is a severe form of hemolytic anemia requiring lifelong regular blood transfusions (1). Prolonged transfusion therapy in TDT patients can trigger the formation of alloantibodies targeting red blood cells (RBCs), leading to alloimmunization (2). The prevalence of alloimmunization in TDT patients ranges widely across studies, ranging from 2.9% to 42.5%, depending on factors such as study sample and transfusion practices (3, 4). Alloimmunization can cause difficulties in finding compatible blood, low transfusion effectiveness, hemolytic transfusion reactions, platelet transfusion refractoriness (PTR), and even life-threatening complications (5).

While previous research is predominantly focused on RBC alloimmunization, the potential risks of platelet alloimmunization are frequently underestimated. Typically, TDT patients have normal platelet counts and do not require platelet transfusions. Nonetheless, procedures such as hematopoietic stem cell transplantation (HSCT) or gene-editing therapy can cause temporary thrombocytopenia, necessitating supportive platelet transfusions (6). Understanding platelet alloimmunization in TDT patients is critical for ensuring effective transfusion therapy and guiding prevention and intervention strategies.

Guangxi province in China has a high prevalence of thalassemia, with carrier frequencies ranging from 20% to 25% (7). Despite this, there is a paucity of research examining the impact of multiple blood transfusions on platelet antibody profiles and the consequent development of PTR in TDT patients. Therefore, this study retrospectively analyzed the prevalence and characteristics of alloimmunization in TDT patients in Guangxi Province between 2014 and 2023, aiming to guide clinical treatment and decision-making for this patient population.

## Methods

### Patients

A total of 257 TDT patients who underwent platelet immunological testing at Nanning Blood Center between January 2014 and December 2023 were enrolled. After excluding patients with a history of splenectomy, prior HSCT, or pregnancy, 249 TDT patients were included in the study. Each patient provided 10 mL of whole blood collected in ethylene diamine tetraacetic acid anticoagulant tubes for platelet antibody testing.

### Platelet antibody testing

Flow cytometry was utilized for platelet antibody testing with the following steps: (i) incubation with the patients’ plasma and platelet antigen panel cells at room temperature for 30 minutes; (ii) washing to remove unbound and non-specifically bound immunoglobulins; (iii) incubation with FITC-(Fab’)2 fragment of goat anti-human IgG (Jackson Immuno Research, USA) for 20 minutes in darkness; (iv) washing to remove unbound fluorescent antibodies. The fluorescence intensity of platelets was measured by the flow cytometer (FACSCanto flow cytometry; BD, USA) and analyzed using BD FACSDiva software.

For participants who have initially tested positive by flow cytometry, enzyme-linked immunosorbent assay (PAKPLUS kit; IMMUCOR, USA) was used for detailed platelet antibody identification with the following steps: (i) coating wells with glycoproteins IIb/IIIa, Ib/IX, Ia/IIa, IV, and human leukocyte antigen(HLA)-Class-I, respectively; (ii) serum incubation at 37°C for 30 minutes; (iii) washing the wells three times; (iv) anti human IgG/A/M conjugate incubation at 37°C for another 30 minutes; (v) adding substrate solution to wells for color development; (vi) adding NAOH solution 30 minutes later to terminate color development. Optical density (OD) was determined using a microplate reader (TECAN, Switzerland). Samples with an OD equal to or greater than two times that of the negative control were considered positive.

### Cross-matching experiment

Cross-matching experiments were conducted on two TDT patients experiencing PTR and who tested positive for HLA antibodies. Potential donor platelets were tested against the patients’ plasma using the platelet antibody detection method outlined earlier to select suitable platelet donors. Transfusion efficiency was assessed using corrected count increment (CCI) (8) calculated as follows:

CCI = increased number of platelets after transfusion (×10^9^) × body surface area (m^2^)/number of platelet transfusions (×10^11^).

A CCI greater than 4.5 for the 24-hour mark suggests a successful transfusion response.

### Statistical analysis

Statistical analysis was conducted using SPSS version 25, with *P < 0.05* indicating statistical significance. Data were presented as numbers (percentages, %) and compared using the chi-square test.

## Results

### Positive rate for platelet antibodies

Among the 249 TDT patients, 103 were detected positive for platelet antibodies (41.37%). **Table 1** compares the positive detection rate for platelet antibodies by age, sex, and blood group, with a significantly higher positive rate observed in patients aged over 12 than those aged under 12 (54.17% vs. 36.16%, *P =* 0.009).

**Table 1:** Comparison of platelet antibody detection rate by age, sex, and blood group.

Characteristics	n	Platelet antibody-positive [n(%)]	χ^2^	*P* value
Age			6.843	0.009
<12 years	177	64 (36.16)		
≥12 years	72	39 (54.17)		
Sex			3.173	0.075
Male	147	54 (36.73)		
Female	102	49 (48.04)		
Blood group			4.513	0.211
A	59	22 (37.29)		
B	64	24 (37.50)		
O	113	54 (47.79)		
AB	13	3 (23.08)		

### Characteristics of platelet antibodies

Among the 103 TDT patients who tested positive for platelet antibodies, 82 (79.61%) exhibited HLA antibodies alone, 15 (14.56%) displayed human platelet antigen (HPA) antibodies alone, while 6 (5.83%) had both HLA and HPA antibodies. Details of antibody subtypes are shown in **Table 2**.

**Table 2:** Characteristics of platelet antibodies in TDT patients.

Type of antibody	Antibody	Positivity of antibody [n(%)]
HLA antibody alone	HLA	82 (79.61)
HPA antibody alone	GP IIb/IIIa	3 (2.9)
	GP Ia/IIa	1 (0.97)
	GP Ib/IX	1 (0.97)
	GP IIb/IIIa+GP Ia/IIa	1 (0.97)
	GP Ia/IIa+GP Ib/IX+GP IV	1 (0.97)
	GP IIb/IIIa+GP Ia/IIa+GP Ib/IX	7 (6.80)
	GP IIb/IIIa+GP Ia/IIa+GP Ib/IX+GP IV	1 (0.97)
Both HLA and HPA antibodies	HLA+GP Ia/IIa	1 (0.97)
	HLA+GP Ib/X	1 (0.97)
	HLA+GP IV	1 (0.97)
	HLA+GP IIb/IIIa+GP Ia/IIa	2 (1.94)
	HLA+GP Ia/IIa+GP Ib/IX	1 (0.97)

### Cross-matching experiment

**Table 3** illustrates the efficacy of cross-matching platelet transfusions in two TDT patients. We observed the platelet counts rose from 0×10^9^/L to 30×10^9^/L in patient 1 and from 3×10^9^/L to 33×10^9^/L in patient 2 after transfusion. Both patients exhibited a 24-hour CCI exceeding 4.5 (10.62 and 7.80, respectively).

**Table 3:** The efficiency of cross-matching platelet transfusion of TDT patients.

Patient	Sex	Platelet counts before transfusion	Platelet counts after transfusion	24 h CCI
1	Male	0×10^9^/L	30×10^9^/L	10.62
2	Female	3×10^9^/L	33×10^9^/L	7.80

CCI, corrected count increment.

## Discussion

Guangxi province is among the regions with the highest prevalence of TDT in China, and alloimmunization poses a significant challenge for TDT patients despite ongoing advancements in blood transfusion protocols (9, 10). This study represents the first effort to analyze the characteristics of platelet antibodies in 249 TDT patients by retrospectively reviewing TDT cases over a decade. Our study showed a high prevalence of platelet alloimmunization in TDT patients (41.37%). Philip et al. found platelet antibodies in 33 out of 80 (41.25%) patients with β thalassemia in western India, which was similar to our study (11). In contrast, a lower prevalence of platelet antibodies (10.26%) was described among multitransfused patients in Algeria (12). The discrepancy may be attributed to variations in antibody testing methodologies and ethnic difference. Additionally, our results also exceeded the previously reported prevalence of RBC alloimmunization in TDT patients in other studies (11.97-18%) (13–16). This phenomenon may be explained by variation in transfusion protocols and diversity in immunogenicity. Prolonged exposure to exogenous antigens through regular transfusions renders TDT patients more susceptible to mounting an immune response. Our findings underscore the critical role of platelet immunity in TDT patients, which warrants clinical and research attention.

This study found a significantly higher detection rate of platelet alloimmunization in TDT patients over 12 years old than in those under 12 years old. This may be explained by the increasing frequency of blood transfusions with advancing age in TDT patients, consequently elevating the risk of immune reactions. HLA antibodies accounted for the largest proportion of platelet antibodies in TDT patients (85.44%). Akahoshi et al. have highlighted that HLA mismatch between donors and recipients is a risk factor for delayed platelet recovery (17). Residual leukocytes in RBC suspensions played a critical role in HLA antibody production, especially in contexts where leukocyte depletion was limited. Additionally, the complex epitope structure of HLA, combined with their high degree of polymorphism, can readily trigger antibody responses in patients. Our findings underscore the potential implications of HLA antibodies not only in post-transfusion reactions but also in subsequent treatment challenges.

Among the 103 TDT patients, 21 were detected positive for HPA antibodies, with most being multiple antibodies (76.19%, 16/21), indicating a propensity for the development of multiple antibodies with long-term transfusion in TDT patients. Anti-HPA-3 exhibited the highest detection rate (66.67%, 14/21), whether in isolation or combination with other antibodies, possibly due to HPA-3 having high heterozygosity in the population of Nanning (18). Notably, we identified antibodies against CD36 in three TDT patients. CD36 deficiency rates vary among populations, reaching 4.13% in China (19), 2.6% in Arabia (20), and 2.14% in Thailand (21). When administering platelet transfusions to TDT patients, it is essential to consider the local population’s HPA distribution characteristics to prevent HPA antibody development.

In this study, platelet cross-matching was conducted on two patients with TDT and PTR, and both demonstrated successful transfusion outcomes. A study conducted in Middle East indicated the number of HLA antibodies as a significant risk factor for PTR in β thalassemia children. The research further demonstrated superior therapeutic efficacy of matched units compared to random units (22). While standard practice for platelet transfusions often involves administering ABO-identical platelets during transfusions, TDT patients, who require frequent transfusions, may benefit from a more specialized approach. The choice of optimal antibody detection and cross-matching strategies for compatible platelet transfusions in PTR patients could be tailored based on local healthcare levels.

This study has several limitations. First, the data were based on one single institution, which may limit the generalizability of our findings. Second, we only included a few variables in the analysis, and there may be other unmeasured variables affecting our results. Retrospective study may not be able to obtain the potential confounding factors of alloimmunization we are interested in which may limit the validity and reliability of the study’s findings. Future multicenter, large-scale studies with robust statistical methods are needed to validate our results.

## Conclusion

This study presents, for the first time, the characteristics of platelet antibodies among patients with TDT in the Nanning region of China. TDT patients demonstrate a high prevalence of platelet alloimmunization, characterized by a diverse array of antibodies, predominantly HLA antibodies. TDT patients should receive leukocyte-depleted RBC suspensions, undergo regular screening for platelet antibodies. For TDT patients with a history of platelet transfusions and positive platelet antibodies, cross-matching can be considered if it is available for favorable financial capacity of patients and sufficient blood resources.

## Data Availability

The raw data supporting the conclusions of this article will be made available by the authors, without undue reservation.
