# Correction: Platelet alloimmunization in transfusion-dependent thalassemia patients from Southern China (2014-2023)

**DOI:** 10.3389/fimmu.2025.1755852

**Published:** 2025-12-03

**Authors:** Frontiers Production Office

**Affiliations:** Frontiers Media SA, Lausanne, Switzerland

**Keywords:** transfusion-dependent thalassemia (TDT), platelet antibody, specificity of antibody, transfusion, cross-matching

Affiliation “PRS Hospital” was erroneously given as “RS Hospital” for reviewer, “Aishwarya MS”.

The original version of this article has been updated.

